# The `Ohana Day Project: A Community Approach to Increasing Cancer Screening

**Published:** 2006-06-15

**Authors:** Kapuaola Gellert, Kathryn L Braun, Robert Morris, Valerie Starkey

**Affiliations:** Papa Ola Lōkahi, `Imi Hale Native Hawaiian Cancer Network; `Imi Hale Native Hawaiian Cancer Network and Professor of Public Health and Social Work, University of Hawaii, Honolulu, Hawaii; Nā Pu`uwai Native Hawaiian Health Care System, Honolulu, Hawaii; Nā Pu`uwai Native Hawaiian Health Care System, Honolulu, Hawaii

## Abstract

**Background:**

Native Hawaiians have higher cancer mortality rates and lower cancer screening rates compared with non-Hawaiians in Hawaii. People living in rural areas have particularly limited options for cancer services, especially for services that are culturally attractive and convenient.

**Context:**

*`Ohana* Day, offered in a small, rural, and predominantly Hawaiian community, was designed to attract underserved Hawaiians to cancer screening.

**Methods:**

The year-long project involved a 1-day *ho`olaule`a* (community celebration) for families that featured 30-minute visits with a same-sex Hawaiian physician (for adults), culturally relevant cancer education and brochures, Hawaiian music, and games for children. Recruitment and follow-up for screening and treatment were offered. Principles of community-based participatory research, Hawaiian values, and Kreuter's strategies guided the design of the event.

**Consequences:**

Of the 73 participants, 10 had abnormal screening results, and all received follow-up screening, treatment, or both within 3 months. Six months after the event, the number of men current with prostate cancer and colorectal cancer screening and the number of women current with clinical breast examination and colorectal cancer screening increased significantly. In addition, the number of participants affiliated with the community's Native Hawaiian health care system and the number with health insurance increased significantly. Participant evaluations showed high overall satisfaction with the *`Ohana* Day program.

**Interpretation:**

Previous studies have noted the barriers to increasing cancer screening among underserved minorities. Culture- and community-based strategies appear to be successful at overcoming these barriers.

## Background

Native Hawaiians are the indigenous people of Hawaii, comprising 20% of the state's population. Other ethnic groups in the state include whites (25%), Japanese (25%), Filipinos (15%), Chinese (5%), and others (1). Compared with whites and Japanese (the state's healthiest and most longevous group), Hawaiians have the highest cancer mortality rates, in part because they often are diagnosed at a late stage after cancer has spread and treatment options are limited (2). For example, 35% of Hawaiian women with breast cancer are diagnosed at a late stage, compared with 29% of whites and 22% of Japanese. Among women with cervical cancer, 41% of Hawaiian women are diagnosed at a late stage, compared with 34% of whites and 30% of Japanese. Among both men and women, 57% of Native Hawaiians with colorectal cancer are diagnosed at a late stage, compared with 50% of whites and 53% of Japanese, as are 33% of Native Hawaiians with melanoma, compared with 9% of whites and 7% of Japanese (2).

Native Hawaiians also have lower rates of cancer screening than other groups. For example, in 2001, only 36% of Hawaiians aged 50 and older had ever had a fecal occult blood test (FOBT), compared with 55% of whites and 55% of Japanese, and only 34% ever had a sigmoidoscopy or colonoscopy, compared with 53% of whites and 56% of Japanese. Only 47% of Hawaiian men aged 40 years and older have ever had a prostate-specific antigen (PSA) test, compared with 56% of white men and 50% Japanese men. Among women, 89% of Hawaiians aged 40 and older have ever had a mammogram compared with 88% of whites and 95% of Japanese (2,3). Hawaiians living in rural areas, especially the islands of Molokai, Lanai, and Niihau, have limited access to cancer screening: none of these islands have capability to provide colonoscopy, and neither Lanai or Niihau have mammography facilities.

**Glossary of Hawaiian terms**
aloha:
compassion
ho`olaule`a:
community celebration
kauka:
physician
kōkua:
proactive helping
kuleana:
acceptance of responsibility and outcomes
laulima:
working together toward a common good
`ohana:
blood relation and extended family
pili:
bonding as family
talk-story:
a form of communication emphasizing personal connections through the sharing of ideas, history, and opinions
`ulu maika:
Hawaiian lawn bowling

Given the low screening rates and late stage of diagnosis, it appears that mainstream cancer screening programs are unsuccessful at recruiting Hawaiians. Other investigators suggest this may be because of the cultural inappropriateness of these programs (4). In contrast, a study conducted in the 1990s found that peer-led outreach groups incorporating traditional Hawaiian values of *kōkua* (proactive helping), *aloha* (compassion), and *pili* (bonding as family) and traditional *talk-story* communications (which emphasize personal connections, respect, active listening, and empathy) increased breast and cervical cancer screening among Hawaiian women (5). The sidebar provides a glossary of Hawaiian terms. Other Hawaiian values relevant to community-based programs are `*ohana* (blood relation and extended family), *kuleana* (acceptance of responsibility and outcomes), and *laulima* (working together toward a common good) (6,7).

Culturally appealing health education materials contribute to successful community-based programs. Kreuter et al recommended five strategies for tailoring materials to minority groups, including the following: 1) peripheral strategies (making materials attractive by including pictures of healthy Hawaiians); 2) evidential strategies (presenting facts about cancer’s impact on Hawaiians); 3) linguistic strategies (using Hawaiian words and phrases); 4) constituent-involving strategies (drawing directly on the experience of Hawaiians affected by cancer), and 5) sociocultural strategies (recognizing and working from Hawaiian values, beliefs, behaviors, and context) (8).

In this project, we tested `*Ohana* (family) Day, a community approach to increase cancer screening, especially among the underserved, that incorporated Hawaiian cultural styles and values. Funding from the Oregon Health & Science University's Native Researchers' Cancer Control Training Program was awarded to a junior researcher of Native Hawaiian ancestry affiliated with *`Imi Hale* — Native Hawaiian Cancer Network, one of the 18 special population networks supported through the National Cancer Institute's (NCI's) Center to Reduce Cancer Health Disparities (9). This paper describes the program, evaluation methods, and lessons learned about the feasibility and effectiveness of this approach.

## Context

The first `*Ohana* Day was held on Molokai, a small island south of Oahu (where 72% of Hawaii's population of 1,200,000 resides). About 80% of Molokai's 7404 residents are Hawaiian. The small island has a relatively low socioeconomic status, with a 12% unemployment rate (compared with 3% to 6% on other islands) (1). During 2000 through 2002, 122 cancer cases were reported on Molokai (Kevin Cassel, Cancer Information Services [CIS], oral communication, January 2005), where each new diagnosis of cancer often has a widespread impact on the members of this close-knit community.

This community was selected because of Molokai's high proportion of medically underserved Hawaiians, the lack of medical resources on the island, and its request for help with cancer screening. Molokai General Hospital, the island's only hospital, is a 30-bed facility providing acute and long-term care and low-risk obstetrical inpatient services. Four family practitioners live on Molokai, an obstetrician–gynecologist is flown in monthly to supervise the midwives and treat patients with complex cases, and other physician specialists visit periodically. Mammography, sigmoidoscopy, and laboratory services are available on the island; colonoscopy is not available.

Many of the island's preventive health services are offered through a partnership between Molokai General Hospital and *Nā Pu`uwai*, a federally funded Native Hawaiian Health Care System (NHHCS). *Nā Pu`uwai* is one of five NHHCSs in Hawaii that provide outreach, access, and primary and secondary prevention services in predominantly Hawaiian communities. About 2000 Molokai residents are registered with *Nā Pu`uwai*, which is staffed by a part-time physician, a registered nurse, four community health workers (CHWs), and a clinical psychologist who visits weekly from Oahu. It has been the practice of *Nā Pu`uwai* and Molokai General Hospital to offer an annual health fair and, at least annually, to have physician specialists from Honolulu fly in to spend a full day seeing patients. However, *`Ohana* Day was the first event to use a festival to draw whole families (rather than individuals only), to feature a large number of Native Hawaiian physicians, and to offer a comprehensive array of primary and secondary cancer screening services.

Because *`Ohana* Day was a research study, we also needed to consider the general distrust of research expressed by many Native Hawaiians following their experience with "hit-and-run" investigations; that is, projects in which researchers extracted data from the community but left no programs or solutions in place (10). Thus, all *`Imi Hale* projects employ community-based participatory research (CBPR) methods, which engage community members in planning, transfer skills, and result in tangible benefits for communities (9-11).

## Methods

*`Ohana* Day, held in October 2003, included the following components: 1) a 1-day *ho`olaule`a* (community celebration and sharing) to which whole families were invited; 2) personalized recruitment by CHWs and the *`Imi Hale* researcher; 3) 30-minute, one-on-one, talk-story–style screening and education visits with a same-sex Hawaiian *kauka* (physician) (physician); 4) culturally relevant cancer education brochures; and 5) follow-up for abnormal findings. Principles of CBPR (10,11), Kreuter's strategies for targeting messages (8), and key Hawaiian cultural values guided the design of this event to address team-identified barriers (Table 1).

Following principles of CBPR, all aspects of *`Ohana* Day, from its development through implementation and dissemination of findings, were undertaken by the *`Ohana* Day team, which included the junior investigator from *`Imi Hale* and two *Nā Pu`uwai* CHWs, with assistance from the *`Imi Hale* program and research directors and several Hawaiian interns. Planning began 6 months before the event, and follow-up activities took an additional 6 months, making this a 1-year project. Decisions about *`Ohana* Day were made by the team at weekly meetings (either in person or by teleconference). These actions helped to ensure that *Nā Pu`uwai* felt ownership in the program and could institutionalize it (10,11). The transfer of skills was bidirectional: *Nā Pu`uwai* staff learned more about research and cancer resources that could be applied to Molokai, and *`Imi Hale* interns learned about community outreach and mobilization strategies from *Nā Pu`uwai* staff.

### Recruitment and registration

An early decision of the research team was to promote *`Ohana* Day through *Nā Pu`uwai*'s existing weekly outreach health screenings and through banners and fliers. During the five weekends preceding *`Ohana* Day, team members from Honolulu assisted team members from Molokai with outreach events held in front of Molokai's major grocery store. To apply the concepts of *pili*, *kōkua*, and *laulima*, the *`Ohana* Day team members, who were known for their practice of Hawaiian cultural values and manners, took time to establish *pili* with community members before suggesting event registration. *`Ohana* Day team members assisted in providing the blood glucose and body fat testing usually offered at outreach events and communicated with participants using talk-story interactions to discover and reinforce personal connections. In this way, Honolulu team members could demonstrate helpfulness and respect for Hawaiian ways and Molokai people, thus gaining credibility.

After the *`Ohana* Day team established trust with participants, it promoted the event according to the Hawaiian concept of *`ohana,* inviting participants to bring whole families, including children. Throughout a talk-story session, the importance of cancer screening, especially for Hawaiians because of their high cancer incidence and mortality rates, was emphasized. *`Ohana* Day team members stressed the *kuleana* of individual Hawaiians to stay healthy for the family and the *kuleana* of family members to take care of each other. The opportunity to spend 30 minutes with a Hawaiian *kauka* (physician) was emphasized, as well as the *ho`olaule`a* (celebratory) nature of the event.

Team members collaboratively devised the registration protocol, and practice sessions were conducted to assure protocol fidelity. The protocol began when a community member decided to attend the event (a decision that may have taken several weeks); the team then assisted him or her through a 30-minute registration process, which included obtaining consent and collecting baseline data on demographic information, *Nā Pu`uwai* membership, insurance status, and screening practices. Participants aged 50 years and older were provided an FOBT kit to complete before the event, and men aged 40 years and older were given laboratory requisitions for PSA testing, both of which were recorded as having been distributed in the baseline questionnaire. This allowed the team to track the participants who would need to discuss results with a *kauka* at the event. Registrants who were not *Nā Pu`uwai* clients were encouraged to sign up and avail themselves of all *Nā Pu`uwai* services, including help with accessing health insurance and providers. We assigned a general timeslot in which to see a physician for each registrant so that we could allocate visits evenly during the 1-day event and minimize participant wait time.

Through the recruitment efforts, we talked to 65 individuals, 62 of whom registered for *`Ohana* Day. We anticipated that some of those recruited would not attend. Because we invited preregistrants to bring other family members, we expected new recruits on the day of the event.

### Brochure development

In priority-setting focus groups conducted in 2000 by *`Imi Hale*, Native Hawaiians reported that available cancer brochures were not relevant to them because they did not feature Hawaiian faces. Thus, since 2000, *`Imi Hale* has undertaken the development of cancer brochures for Native Hawaiians with content from NCI and American Cancer Society Web sites, message and format recommendations from key informants and focus groups, and consumer pretesting. The following materials were made available at *`Ohana Day*: 1) a five-booklet series on breast cancer (12), 2) a colorectal cancer brochure (13), 4) a smoking cessation brochure (9), and 5) draft versions of brochures on oral, prostate, testicular, cervical, and skin cancers (14). In line with Kreuter's strategies (8), *`Imi Hale* brochures feature healthy Hawaiian faces, acknowledge Hawaiian values, and include testimonials from Hawaiian peers, health promotion messages from Hawaiian physicians, and information about the impact of cancer on Hawaiians (Table 1).

### Event day

As with all aspects of *`Ohana* Day, team members collaboratively designed protocol related to the day of the event, and volunteers (including other staff and *kauka*) were trained the day before. Participants signed in, and those who had not preregistered spent their first 30-minutes being assisted with the consent form and baseline questionnaire. Participants were then moved through a series of stations, starting with Cancer 101, a basic cancer education session developed in collaboration with Hawaii's Cancer Information Service (CIS) and presented by a *kauka* who emphasized the importance of early cancer detection and treatment for Hawaiians. Adults then met one-on-one with a same-sex *kauka* for 30 minutes for screening and education. The 15 *kauka* that volunteered on event day were members of the *`Ahahui O Nā Kauka* (Association of Native Hawaiian Physicians). They arrived on the island the previous evening, allowing time to become oriented to the event and to provide a free continuing medical education workshop on cancer among Native Hawaiians. The project paid for travel and on-island accommodations for the *kauka*, and the *kauka* donated their time to the project. During their stay on Molokai, the *kauka* were seen in casual clothing and in friendly interactions to help participants feel comfortable.

From female *kauka*, women received a clinical breast examination, instruction in breast self-examination, and education and screening for oral and skin cancers. From male *kauka*, men received education and screening for skin, oral, prostate, and testicular cancers. Although the U.S. Preventive Services Task Force does not recommend routine screening for oral (15), skin, prostate, or testicular cancers, we offered the examinations at *`Ohana* Day because they allowed participants to spend more time, often more than 30 minutes, with *kauka*, increase their symptom-detection knowledge, and increase their comfort with talking about cancer. Additional health education stations focused on site-specific cancers and lifestyle behaviors and included a station staffed by a CIS member devoted to skin cancer education and at which sunscreen samples were distributed. An Ask-a-*Kauka* station allowed participants to ask a *kauka* any medical questions. Education and screening concluded with a personalized exit interview with a *kauka* who emphasized *kuleana* in caring for oneself and others and summarized recommendations for follow up, future screening (including mammograms, colonoscopies, and Papanicolaou tests [Pap smears]), and lifestyle changes (such as smoking cessation, improved diet, and more exercise).

Participants were served a healthy Hawaiian lunch, and music was performed by popular local musicians. Children attending the event participated in Hawaiian games, such as *`ulu maika* (Hawaiian lawn bowling), and education sessions on healthy eating, exercising, using sunscreen, and not smoking. Finally, adult participants completed a self-administered survey (at the fifth-grade reading level) with an item on satisfaction scored on a 5-point Likert-type scale and four open-ended items on what participants learned, liked best, would recommend to improve the event, and wanted to learn about in future programs.

### Follow-up activities

As defined in the team-developed protocol for follow up, within a month of the event, participants received letters explaining their screening results, reiterating recommendations for follow up on abnormal findings, and recommending screenings not provided at the event. Assistance with obtaining insurance, scheduling appointments, and transportation also was offered in the letter. Up to 6 months thereafter, two members of the *Nā Pu`uwai* staff — a male staff member for male participants and a female staff member for female participants — made weekly phone calls to participants to encourage compliance with recommended screening and diagnostic tests.

### Statistical methods

SPSS (SPSS Inc, Chicago, Ill) was used to manage and analyze data. The two-tailed exact test version of McNemar's test was used to determine the significance of changes in measures of health care status from event registration to 6 months after the event. The measures included whether participants were affiliated with *Nā Pu`uwai*, had health insurance, had seen a physician in the past 5 years, and were current with cancer screening examinations.

## Consequences

On the day of the event, 73 adults from 41 families were screened, including 63 Native Hawaiians, eight Filipinos, and two whites. The 73 participants included 42 who had preregistered. Of the 20 who preregistered but did not attend, we later learned that 11 attended funerals of two community members who had recently died and nine attended a community youth baseball game. Of the 31 who registered on event day, five were men who accompanied their wives to the event and were persuaded to participate while waiting for their wives, 14 attended with family members who had preregistered, and 12 had learned of the event through friends and neighbors.

Table 2 compares measures of health care status of participants before and after the event. Based on registration data for the 73 adult participants, 23 (32%) were not *Nā Pu`uwai* clients, 11 (15%) were uninsured, and five (7%) had not seen a physician in 5 or more years. One 55-year-old man reported never having had a physical examination. Only 39% of the men aged 50 and older were current with colorectal cancer screening (either FOBT, sigmoidoscopy, or colonoscopy), and 39% of men aged 40 and older were current with prostate cancer screening. For women, only 36% of those aged 50 and older were current with colorectal cancer screening. About one third of women aged 40 and older were not current with breast cancer screening, including 13 women who had never had a clinical breast examination or mammogram.

Significant improvements were found in screening compliance (whether received at the event or in the following 6 months) and insurance coverage 6 months after the event. For example, at 6-month follow up, 76% of female participants aged 50 years or older were current with colorectal cancer screening, 84% aged 40 years or older with mammogram, and 100% aged 40 years or older with clinical breast examination screening. For men, 97% aged 40 years or older were current with prostate cancer screening, and 75% aged 50 years or older were current with colorectal cancer screening. All 10 participants with abnormal findings (one colorectal, two breast, two prostate, and five skin) received follow-up screening, treatment, or both within 3 months of the event. All 23 participants who were not previous *Nā Pu`uwai* clients became clients, and all 11 without health insurance received assistance obtaining health insurance. Because insurance enrollment is a time-consuming process, free follow up was obtained for five uninsured participants who needed immediate attention for abnormal findings. Since the end of the project, 71 of 73 participants and their families have continued to seek assistance from *Nā Pu`uwai* with cancer screenings and other health issues, including hypertension and diabetes control (Valerie Starkey, unpublished data, November 2005).

In evaluating *`Ohana* Day, 71 (93%) participants gave it the highest satisfaction rating on a 5-point scale. Best liked were education, including the brochures targeting Native Hawaiians (n = 26), everything (n = 17), one-on-one visits with the *kauka* (n = 16), and inclusion of family (n = 6). Participants were impressed with the helpfulness and caring of the *kauka* and the CHWs. One participant commented, "If they care about my health, I should too." When asked what should be changed, 25 participants said "nothing," and three said, "less waiting time to see the doctor." Participants wanted to learn more about such topics as diabetes, obesity, cardiovascular disease, psychology, and asthma.

## Interpretation

As noted in previous studies, barriers to increasing cancer screening among racial and ethnic minority populations include limited cancer screening knowledge, limited accessibility of health services (especially culturally tailored services), and lack of clinician recommendations (4,5,11,16,17). *`Ohana* Day employed culture- and community-based strategies to address these barriers, and evaluation data suggest that *`Ohana* Day appealed to Hawaiians on Molokai and was effective at increasing cancer screening rates and bringing people without insurance, a medical point of contact, or both into the health care system.

A key element of success was *`Ohana* Day's fit with the community, which was ensured by joint planning and implementation by `Imi Hale and Nā Pu`uwai as equal partners, in contrast to many projects, which are designed by outsiders and modified through community involvement. Other elements of success were the time devoted to personalize recruitment, event day, and follow-up and the demonstration of caring by kauka and CHWs. These elements helped reduce the chance of participants dropping out during the year. The elements also helped establish and enhance relationships among community members and health providers, which proved necessary for changing health care use patterns among the underserved. The power of trust building also was reflected in the fact that 26 individuals registered for screening on event day, citing the recommendation of a family member or friend.

Also critical was the cultural targeting of Cancer 101 and brochures on cancer screening; *`Imi Hale* continues to develop and disseminate cancer-related brochures and curricula (9,13,14). Although not mentioned in the written evaluations by participants, both participants and providers expressed appreciation for brochures with Hawaiian faces. That 16 individuals indicated that they liked "everything" suggests that the relaxed, festival-like atmosphere was also important. Although each participant was allowed 30 minutes or more with a *kauka* and some participants had to wait to be seen, only three participants mentioned the waiting time as the thing they liked least.

We were gratified that the event attracted 23 more community members to avail themselves of *Nā Pu`uwai* services because our intent was to reach the medically underserved. Among the 50 participants who already were clients of *Nā Pu`uwai*, we were not surprised to find that many were not current with cancer screening recommendations, especially the men. Subsequent focus groups with Native Hawaiian men found that they avoid visiting physicians unless they are in pain (18). Men also communicated that health was traditionally the *kuleana* of women, and so they depended on wives and daughters to pressure them to seek health care when needed. This observation underscores the relevance of an *`ohana*-targeted event in Native Hawaiian communities; that is, increased awareness among women may help increase screening compliance for the entire family.

Despite intensive follow-up, one man did not have a PSA test within 6 months of the event, 6 women did not have mammograms, and 13 women did not have colorectal cancer screening. It was not surprising that postintervention colorectal cancer screening rates did not reach 100% because Molokai does not have colonoscopy capabilities and, in a separate study by *`Imi Hale* to increase colorectal cancer screening rates, we found that postintervention rates did not exceed 85% (13). Because all *`Ohana* Day participants are registered with *Nā Pu`uwai*, however, staff continue to remind them about their need for regular cancer screening.

*`Ohana* Day proved feasible on Molokai, and *Nā Pu`uwai* has incorporated *`Ohana* Day as an annual event. In 2004, the *Nā Pu`uwai* staff organized a similar cancer screening and education program on another small, medically underserved Hawaiian island (Lanai), with limited assistance from the *`Imi Hale* staff. These developments suggest that the Molokai staff has the experience and confidence to use the established protocol and to call on the services of the *`Ahahui O Nā Kauka* and others to continue the program on these two islands.

*`Ohana* Day's success and acceptance has led to several requests to offer it in other communities, but the question is whether it would be feasible. Certainly the time-intensive nature of *`Ohana* Day requires that it be prioritized for medically underserved communities with limited resources, rather than in communities with access to an array of cancer screening services.

Small sample size was a limitation to our study. Although many participants registered on-site, about 20 community members who had preregistered did not attend the event because of other obligations. Also, because a major goal was to test the feasibility of the event, we only recruited participants, and there was no control group against which to measure our effectiveness. Next steps include seeking funds for an experimental test of the program in other underserved Hawaiian communities, using a randomized controlled design, to examine its effectiveness with increasing cancer screening rates and bringing people without insurance, a medical point of contact, or both into the health care system. Given the participant appreciation of the educational aspects of the intervention, we recommend adding questionnaires to assess knowledge and attitudes to evaluations of future offerings. Future research also should be performed to identify barriers to cancer screening in Native Hawaiian communities.

Despite limitations, preliminary data suggest that interventions such as *`Ohana* Day that employ culture- and community-based strategies can increase compliance with cancer screening and follow-up recommendations in underserved Hawaiian communities.

## Figures and Tables

**Table 1:** Hawaiian Concepts and Kreuter Strategies Incorporated to Increase Cancer Screening

**Problem**	**Solution**	

	**Hawaiian Concepts**	**Kreuter Strategy^a^**
Programs and materials are not attractive to Hawaiians.	Native Hawaiians helped (*kōkua*) design *`Ohana* Day.	Constituent-involving
	*`Ohana* Day was a family-focused (*`ohana*), all-day community celebration (*ho`olaule`a*), featuring Hawaiian music and food, and balancing (*Lōkahi*) health information and screening with fun.	Sociocultural
	Health education materials were developed, featuring healthy Hawaiians and Hawaiian words and phrases.	Peripheral, linguistic
	A presentation on cancer by a Native Hawaiian physician included evidence that cancer is a problem for Native Hawaiians.	Evidential
Some Native Hawaiians are distrustful of or uncomfortable with mainstream health services and providers.	Trusting and helping relationships (*pili*, *kōkua*) between the community, clinic staff, and researchers were built before the event took place.	Constituent-involving and sociocultural
	Participating Native Hawaiian physicians (*kauka*) were seen in casual clothing and in friendly interactions. Participants could talk leisurely with a Native Hawaiian physician (e.g., during lunch) and spend up to an hour with the participant during one-on-one screening.	Sociocultural
	Native Hawaiian physicians (*kauka*) reviewed findings and prescribed follow-up on abnormal findings, completion of screenings not provided at the event, and relevant lifestyle changes. Responsibility (*kuleana*) for health was stressed.	Sociocultural
	The community was asked to help (*kōkua*) critique the event.	Constituent-involving
Some Native Hawaiians put caring for others before caring for self.	Recruiters and physicians explained that people must care for themselves to be healthy enough to honor family roles and responsibilities (*`ohana*, *kuleana*, *kōkua*).	Sociocultural
	Clinic staff scheduled follow-up care and screening, mailed follow-up letters, and telephoned participants to encourage action (*pili*, *kōkua*).	Sociocultural
Unscreened individuals do not come to the health center.	Native Hawaiian researchers and clinic staff went into the community to recruit individuals; they sat in front of grocery stores for 5 weekends before the event to build interest and trust (*pili*).	Constituent-involving and sociocultural

a Source: Kreuter et al (8).

**Table 2:** Comparison of Preintervention and 6-Month Postintervention Health Care Status Among Participants in *`* *Ohana* Day, Molokai, Hawaii, October 2003

**Health Care Status**	**No. of Participants**	**Preintervention No. (%)**	**Postintervention No. (%)**	***P* Value^a^**
**Affiliated with *Nā Pu`uwai***	73	50 (68)	73 (100)	<.001
**Has health insurance**	73	62 (85)	73 (100)	<.001
**Has seen a doctor within 5 y**	73	68 (93)	73 (100)	.06
**Cancer screening examination**				
Prostate cancer screening among men aged ≥40 y	33	13 (39)	32 (97)	<.001
Colorectal cancer screening among men aged aged ≥50 y	28	11 (39)	21 (75)	.002
Clinical breast examination among women aged ≥40 y	38	25 (66)	38 (100)	<.001
Mammogram among women aged ≥40 y	38	25 (66)	32 (84)	.02
Colorectal cancer screening among women aged ≥50 y	25	9 (36)	19 (76)	.002

a The two-tailed exact test version of McNemar's test was used to check for significance in changes in the number of participants.
